# A novel combined neutrophil-platelet score for diagnosing complicated appendicitis: a retrospective and prospective cohort study

**DOI:** 10.1590/acb413926

**Published:** 2026-07-24

**Authors:** Xiang Li, Jiangshun Yang, Dengyang Fang, Wei Dai, Zuming Xiong, Xuan Chen

**Affiliations:** 1 Chongqing University – Chongqing University Fuling Hospital – Department of Gastrointestinal Surgery – Chongqing – China.; 2 Chongqing University – School of Medicine – Chongqing – China.

**Keywords:** Appendicitis, Biomarkers, Severity of Illness Index, Neutrophils, Platelet Count

## Abstract

**Purpose:**

We investigated the neutrophil-to-lymphocyte ratio (NLR) and the platelet-to-lymphocyte ratio (PLR) and combined them to create an inflammatory marker, the combined neutrophil-platelet score (CNPS), to serve as a diagnostic and predictive tool for complicated appendicitis.

**Methods:**

A retrospective analysis was conducted on the clinicopathological data of patients who underwent appendectomy. Receiver operating characteristic (ROC) curves and the area under the curve (AUC) were used to evaluate the diagnostic performance of various inflammatory parameters. Univariate and multivariate logistic regression analyses were performed to identify independent risk factors for complicated appendicitis. A prospective study was then carried out to validate the clinical utility of the CNPS.

**Results:**

In total, 322 patients were included in the retrospective analysis. Multivariate analysis identified only onset time and NLR as independent risk factors for complicated appendicitis, whereas the PLR was not. CNPS demonstrated superior diagnostic and predictive performance for complicated appendicitis compared to NLR and PLR. Moreover, after adjusting for confounders, CNPS remained an independent risk factor for complicated appendicitis. In the prospective study, 264 patients were included for analysis, and the AUC of CNPS for predicting complicated appendicitis was 0.707.

**Conclusion:**

The CNPS, combining NLR and PLR, demonstrates high diagnostic and predictive value for complicated appendicitis.

## Introduction

Acute appendicitis is the fourth most common cause of acute abdominal pain^1^, frequently presenting as a cause of acute lower abdominal pain and prevalent in young patients^2^. According to the 2020 guidelines from the World Society of Emergency Surgery, acute appendicitis is classified into uncomplicated and complicated types. Uncomplicated appendicitis (UA) includes simple and septic appendicitis. In contrast, complicated appendicitis (CA) is characterized by gangrene, perforation of the appendix from the tip to the root, and periappendiceal abscess formation^2-4^. Gangrenous and perforated appendicitis represent severe forms of the condition that, if untreated, can lead to postoperative complications and increased mortality. Acute uncomplicated appendicitis has a mortality rate of less than 0.1%, gangrenous appendicitis about 0.6%, while perforated appendicitis can result in a mortality rate of approximately 5%^2^.

Since its inception in the 19th century, appendectomy has become a widely accepted surgical procedure^5^. With advancements in surgical diagnostics and treatment techniques, laparoscopic appendectomy is nowadays considered the most effective treatment^6,7^. This approach offers benefits such as shorter operative time, less intraoperative bleeding, fewer postoperative complications, and reduced hospitalization time. However, it has been observed that some patients with clinically diagnosed acute appendicitis, who are indicated for emergency surgery, refuse the procedure due to personal reasons. Consequently, some of these patients experience poor prognoses due to the continued progression of the disease.

Several scoring systems have been developed to aid in the diagnosis of acute appendicitis, including the Alvarado score^8^, the Appendicitis Inflammatory Response score^9^, and the Raja Isteri Pengiran Anak Saleha score^10^. However, these systems have poor specificity and sensitivity and do not assess the severity of appendicitis, meaning they cannot distinguish between UA and CA.

Complicated appendicitis, if left undiagnosed and untreated, may lead to serious complications such as total peritonitis and infectious shock, which are difficult to treat and have a high mortality rate. Therefore, finding a suitable tool or marker to help clinicians differentiate between UA and CA is crucial. Both neutrophil-to-lymphocyte ratio (NLR) and platelet-lymphocyte ratio (PLR) are inexpensive and easily accessible markers of inflammation that can be calculated from routine blood tests^11,12^. However, the predictive value of NLR and PLR in complicated appendicitis remains controversial in clinical practice. Thus, this study aimed to explore whether different clinical types of appendicitis have distinct NLR and/or PLR levels, and determine if a combination of NLR and PLR is more accurate in predicting CA than either marker alone.

## Methods

### Ethics approval statement

This study was conducted in accordance with the Declaration of Helsinki (revised 2013). The study was reviewed and approved by the Ethics Committee of Chongqing University Fuling Hospital (2024CDFSFLYYEC-023).

### Retrospective study

#### Study population and inclusion criteria

The clinicopathological data of patients who were hospitalized in the Department of Gastrointestinal Surgery and underwent appendectomy at Chongqing University Fuling Hospital, between January 1, 2021, and September 30, 2023, were retrospectively analyzed. All patients were pathologically diagnosed (gold standard) with acute appendicitis after surgery. The age range of the patients was greater than 16 years old and less than 65 years old.

#### Exclusion criteria

The exclusion criteria were: systemic immune diseases, hematologic diseases, acute and chronic infections, hepatic and renal insufficiency; a history of blood transfusion or gastrointestinal hemorrhage within the past month; recently used hormones, anti-inflammatory, or anti-platelet drugs; and incomplete clinicopathologic information.

#### Data collection

Blood sampling and the collection of relevant inflammatory markers were completed within 24 hours of admission for patients diagnosed with acute appendicitis and scheduled for surgical intervention^13^. A comprehensive approach was used in data collection. The patients were categorized into two groups: UA and CA. The collected data included baseline information (gender, age, onset time, peritonitis, smoking status, and alcohol intake) and results from the first post-admission or pre-admission outpatient emergency routine blood tests (neutrophil count, lymphocyte count, and platelet count). Peritonitis was assessed as follows:

- No signs of peritonitis: only right lower abdominal pressure without rebound pain and muscle tension;
- Right lower abdominal peritonitis: right lower abdominal pressure, rebound pain, and muscle tension;
- Lower abdominal peritonitis: pressure, rebound pain, and muscle tension in the right lower, lower-middle, and lower-left abdomen;
- Total peritonitis: pressure, rebound pain, and muscle tension throughout the abdomen.

The NLR was calculated as Eq. 1:


(1) NLR=neutrophilcount(×109/L)/lymphocytecount(×109/L)

The PLR was calculated as Eq. 2:


(2) PLR=plateletcount(×109/L)/lymphocytecount(×109/L)

Data for each patient were independently extracted by two team members. Any disagreements were resolved through discussion between the members or, if necessary, by consulting a third independent member.

### Prospective study

#### Study population and inclusion criteria

Hospitalized patients diagnosed with acute appendicitis and scheduled to undergo appendectomy in the Department of Gastrointestinal Surgery of our hospital were consecutively recruited into the prospective clinical study between January 1, 2024, and May 31, 2026. Eligible patients were between 16 and 65 years old and had signed a written informed consent form for participation in the study.

#### Exclusion criteria

Patients were excluded from the study if they met any of the following conditions:

- Presence of underlying systemic autoimmune diseases, hematological disorders, acute or chronic infections, or hepatic/renal insufficiency;
- History of blood transfusion or major gastrointestinal bleeding within the past month;
- Recent use of corticosteroids, anti-inflammatory agents, or antiplatelet medications.

#### Diagnostic criteria for acute appendicitis

The clinical diagnosis of acute appendicitis was based on the following criteria: abdominal pain, with or without migration to the right lower quadrant; presence or absence of gastrointestinal symptoms such as nausea and vomiting; localized tenderness in the right lower abdomen, with or without rebound tenderness and muscle guarding; leukocytosis in the complete blood count, which may or may not be present; and imaging findings (ultrasound or computed tomography) showing an enlarged appendix and/or periappendiceal fluid or fat stranding. The duration of symptoms was ≤ 72 hours.

#### Complete blood count and pathological examination

Upon admission, all enrolled patients had 2 mL of peripheral venous blood drawn for complete blood count testing. The measured parameters included neutrophils, monocytes, and lymphocytes, using the XN-10[B4] hematology analyzer. The operating surgeons were blinded to the patient’s enrollment status in the study. During surgery, the resected appendix specimen was immediately fixed in 4% formalin and sent for histopathological examination. The pathologists were blinded to the patients’ laboratory results and intraoperative findings, unless diagnostic clarification was necessary. In cases in which the postoperative clinical diagnosis differed from the pathological diagnosis, discrepancies were resolved through discussion between the operating surgeon and the pathologist.

#### Withdrawal criteria

Patients were excluded from the final analysis if they met any of the following conditions:

- Postoperative diagnosis was not appendicitis (*e.g.*, appendiceal tumor);
- The patient declined to undergo surgery after enrollment;
- The patient withdrew from the study during participation.

### Data analysis

Receiver operating characteristic (ROC) curves and the area under the curve (AUC) were used to assess the diagnostic performance of the inflammatory parameters^14^. The optimal cut-off value for each parameter was determined by maximizing Youden’s index (sensitivity + specificity - 1)^15^. Data were then divided into two groups (high and low inflammation) based on the cut-off values, and differences in clinical characteristics between the two groups were compared. The performance of inflammation parameters in predicting CA was evaluated by assessing sensitivity, specificity, positive predictive value, negative predictive value, and their respective 95% confidence intervals (95%CI). Univariate and multivariate logistic regression analyses were conducted to identify independent risk factors for CA. Variables that were clinically relevant or showed potential differences between groups were included in the multivariate model to adjust for potential confounding.

For continuous variables, the mean (standard deviation) was used for description. Frequencies and percentages were used for categorical variables. Independent samples t-test (Student’s test) was used for between-group comparisons of continuous variables that followed a normal distribution, while the Mann-Whitney’s U test was used for those that did not. For between-group comparisons of categorical variables, the χ^2^ test or Fisher’s exact test was used. Of note, no hypothesis tests were performed for comparisons of baseline characteristics between uncomplicated and complicated appendicitis groups, as such tests are not meaningful in this observational study design. A p-value of less than 0.05 (two-tailed) was considered statistically significant. Statistical analysis was performed using R software (version 4.2.2).

## Results

**Figure 1:**
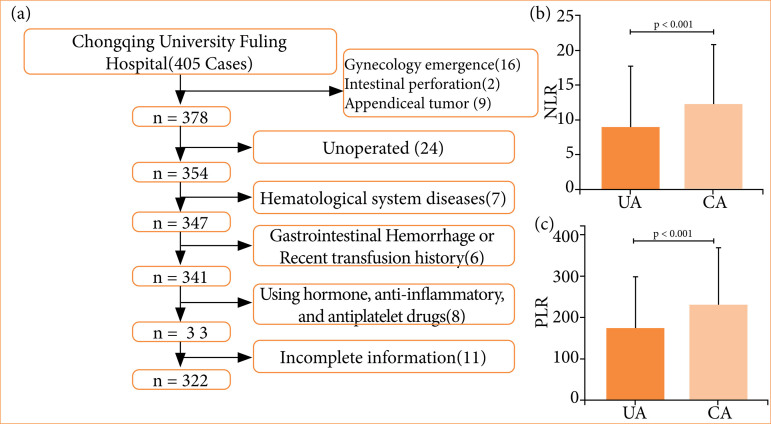
Data screening and bar chart showing complicated appendicitis (CA) patients (N = 235) and uncomplicated appendicitis (UA) patients (N = 87) had significantly different inflammatory status (*p* < 0.001). (A) Flowchart showing patients screening process. (B) Neutrophil-to-lymphocyte ratio was significantly higher in CA patients than UA patients. (C) Platelet-lymphocyte ratio was significantly higher in CA patients than in survivors.

Source: Elaborated by the authors.

**Figure 2:**
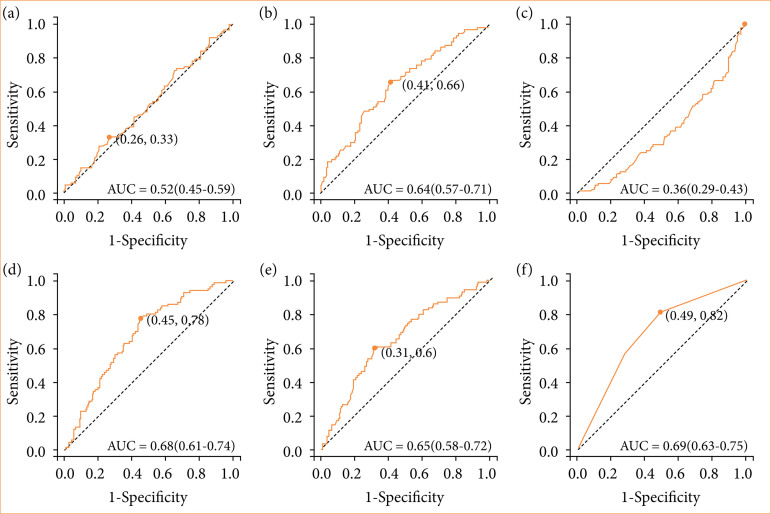
Receiver operating characteristic curves demonstrating the ability of each inflammatory parameter to diagnose and predict complicated appendicitis. (A) Platelet; (B) neutrophils; (C) lymphocyte; (D) neutrophil-to-lymphocyte ratio; (E) platelet-lymphocyte ratio; (F) combined neutrophil-platelet score.

We retrospectively analyzed the medical records of 405 patients diagnosed with acute appendicitis who underwent appendectomy in the Department of General Surgery at Chongqing University Fuling Hospital, from January 1, 2021, to September 30, 2023. Based on our inclusion and exclusion criteria, data from 322 patients were included in the final analysis (Fig. 1a). Among them, 235 cases were classified as UC and 87 cases as CA. Baseline characteristics were compared between the UA and CA groups. Patients with CA were generally older, had a longer onset time, and showed higher neutrophil count and lower lymphocyte count compared with those with UA (Table 1). Both the NLR and the PLR were higher in the CA group compared to the UA group, with the differences being statistically significant (Figs. 1b and 1c). We plotted ROC curves for each parameter and calculated the AUC to evaluate the diagnostic ability of each inflammatory parameter for CA. Among them, neutrophil count (AUC = 0.639, 95%CI 0.57–0.71), NLR (AUC = 0.675, 95%CI 0.61–0.74), and PLR (AUC = 0.652, 95%CI 0.59–0.72) demonstrated better predictive ability (Fig. 2, Table 2). Notably, NLR exhibited the highest predictive value with an AUC of 0.675 (*p* < 0.001). Table 2 also presented the best cut-off value, sensitivity, and specificity for each parameter.

**Table 1:** Baseline characteristics of patients with acute appendicitis<tfn href="tfn01">*</tfn>.

Characteristics	Overall (n = 322, %)	UA (n = 235, %)	CA (n = 87, %)
Gender			
Female	169 (52)	125 (53)	44 (51)
Male	153 (48)	110 (47)	43 (49)
Age (mean, SD)	41 (13)	40 (13)	45 (13)
Onset time (mean, SD)	22 (17)	20 (15)	29 (18)
Peritonitis			
No	56 (17)	43 (18)	13 (15)
Right lower abdomen	235 (73)	174 (74)	61 (70)
Lower abdomen	15 (4.7)	7 (3)	8 (9.2)
Whole abdomen	16 (5)	11 (4.7)	5 (5.7)
Smoking status			
Nonsmoker	250 (78)	176 (75)	74 (85)
Previous smokers	52 (16)	43 (18)	9 (10)
Occasional smokers	9 (2.8)	7 (3)	2 (2.3)
Regular smokers	11 (3.4)	9 (3.8)	2 (2.3)
Alcohol intake			
Nondrinker	250 (78)	177 (75)	73 (84)
Previous drinkers	14 (4.3)	13 (5.5)	1 (1.1)
Occasional drinkers	55 (17)	43 (18)	12 (14)
Regular drinkers	3 (0.9)	2 (0.9)	1 (1.1)
PLA (mean, SD)	217 (61)	215 (59)	223 (68)
NEU (mean, SD)	10.4 (3.8)	9.9 (3.7)	11.7 (3.8)
LYM (mean, SD)	1.52 (0.84)	1.62 (0.87)	1.25 (0.67)
NLR (mean, SD)	10 (9)	9 (9)	12 (9)
PLR (mean, SD)	190 (128)	175 (121)	231 (138)

UA: uncomplicated appendicitis; CA: complicated appendicitis; SD: standard deviation; PLA: platelet; NEU: neutrophils; LYM: lymphocytes; NLR: neutrophil‐to‐lymphocyte ratio; PLR: platelet-lymphocyte ratio;

* no formal hypothesis testing was performed for baseline comparisons.

Source: Elaborated by the authors.

AUC: area under the curve. Source: Elaborated by the authors.

**Table 2:** Assessment of the ROC characteristic parameters of each biomarker.

Name	Cutoff	AUC	p-value	95%CI	SEN	95%CI	SPE	95%CI	PPV	95%CI	NPV	95%CI
PLA	248.5	0.52	0.7	0.45– 0.59	0.33	0.23– 0.43	0.74	0.68– 0.79	0.32	0.22– 0.41	0.75	0.69– 0.81
NEU	10.65	0.64	< 0.001	0.57– 0.71	0.66	0.56– 0.76	0.59	0.52– 0.65	0.37	0.29– 0.45	0.82	0.76– 0.88
LYM	0.31	0.36	> 0.99	0.29– 0.43	> 0.99	1–1.000	0.01	-0.003– 0.02	0.27	0.22– 0.32	> 0.99	1–1
NLR	6.45	0.68	< 0.001	0.61– 0.74	0.78	0.69– 0.87	0.55	0.49– 0.61	0.39	0.32– 0.46	0.87	0.82– 0.93
PLR	177.4	0.65	< 0.001	0.59– 0.72	0.59	0.49–0.7	0.68	0.63– 0.74	0.41	0.33– 0.49	0.82	0.77– 0.88
CNPS	0.5	0.69	< 0.001	0.63– 0.75	0.82	0.74– 0.89	0.51	0.45– 0.58	0.38	0.31– 0.45	0.88	0.83– 0.94

PLA: platelet; NEU: neutrophils; LYM: lymphocyte; NLR: neutrophil‐to‐lymphocyte ratio; PLR: platelet-lymphocyte ratio; CNPS: combined neutrophil-platelet score; AUC: area under the curve; SEN: sensitivity; SPE: specificity; PPV: positive predictive value; NPV: negative predictive value; 95%CI: 95% confidence interval. Source: Elaborated by the authors.

To investigate the relationship between NLR and PLR and CA, we categorized the values into high and low groups based on their optimal cutoffs and then assessed the clinical characteristics of each group. We observed that the high NLR group had a significantly higher proportion of CA cases and older patients (*p* < 0.05) (Table 3). Similarly, the high PLR group not only had a higher percentage of CA cases and older individuals but also exhibited a higher incidence of lower abdominal and total peritonitis, as well as a notable history of regular smoking (Table 3).

**Table 3:** The association between inflammatory biomarkers and clinical features.

Characteristic	Overall (N = 322)	NLR		*p* -value	PLR		*p* -value	CNPS		*p* -value
		High NLR (N = 174)	Low NLR (N = 148)		High PLR (N = 126)	Low PLR (N = 196)		High CNPS (N = 186)	Low CNPS (N = 136)	
Group				< 0.001			< 0.001			< 0.001
UA	235 (73)	106 (61)	129 (87)	74 (5٪)	161 (82)	115 (62)	120 (88)			
CA	87 (27)	68 (39)	19 (13)		52 (41)	35 (18)		71 (38)	16 (12)	
Gender				0.10			> 0.9			0.4
female	169 (52)	84 (48)	85 (57)		66 (52)	103 (53)	94 (51)	75 (55)		
male	153 (48)	90 (52)	63 (43)		60 (48)	93 (47)		92 (49)	61 (45)	
Age (mean, SD)	41 (13)	44 (13)	38 (13)	< 0.001	43 (13)	40 (13)	0.009	44 (13)	38 (12)	< 0.001
Onset.time (mean, SD)	22 (17)	21 (17)	23 (17)	0.2	23 (17)	22 (17)	0.3	22 (17)	23 (17)	0.7
Peritonitis				0.061			0.006			0.028
No	56 (17)	34 (20)	22 (15)		26 (21)	30 (15)		37 (20)	19 (14)	
Right lower abdomen	235 (73)	118 (68)	117 (79)	81 (64)	154 (79)	126 (68)	109 (80)			
Lower abdomen	15 (4.7)	9 (5.2)	6 (4.1)		7 (5.6)	8 (4.1)		9 (4.8)	6 (4.4)	
Whole abdomen	16 (5.0)	13 (7.5)	3 (2.0)		12 (9.5)	4 (2.0)		14 (7.5)	2 (1.5)	
Smoking status				0.2			0.030			0.2
Nonsmoker	250 (78)	134 (77)	116 (78)	100 (79)	150 (77)	146 (78)	104 (76)			
Previous smokers	52 (16)	28 (16)	24 (16)		17 (13)	35 (18)		28 (15)	24 (18)	
Occasional smokers	9 (2.8)	3 (1.7)	6 (4.1)		1 (0.8)	8 (4.1)		3 (1.6)	6 (4.4)	
Regular smokers	11 (3.4)	9 (5.2)	2 (1.4)		8 (6.3)	3 (1.5)		9 (4.8)	2 (1.5)	
Alcohol intake				0.5			0.054			0.2
Nondrinker	250 (78)	139 (80)	111 (75)	103 (82)	147 (75)	150 (81)	100 (74)			
Previous drinkers	14 (4.3)	5 (2.9)	9 (6.1)		1 (0.8)	13 (6.6)		5 (2.7)	9 (6.6)	
Occasional drinkers	55 (17)	29 (17)	26 (18)		21 (17)	34 (17)		30 (16)	25 (18)	
Regular drinkers	3 (0.9%	1 (0.6)	2 (1.4)		1 (0.8)	2 (1.0)		1 (0.5)	2 (1.5)	

UA: uncomplicated appendicitis; CA: complicated appendicitis; SD: standard deviation; NLR: neutrophil-to-lymphocyte ratio; PLR: platelet-lymphocyte ratio; CNPS: combined neutrophil-platelet score. Source: Elaborated by the authors.

**Figure 3:**
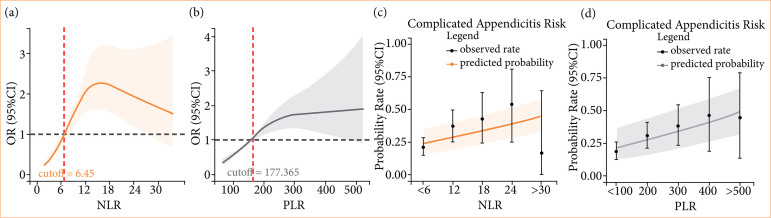
The relationship between inflammatory parameters and complicated appendicitis (CA) was evaluated. The OR and 95%CI of CA were evaluated based on the baseline levels of (a) NLR and (b) PLR. The predicted likelihood of CA versus the actual rate was evaluated based on the baseline levels of (c) NLR and (d) PLR.

NLR: neutrophil-to-lymphocyte ratio; PLR: platelet-lymphocyte ratio; OR: odds ratio; 95%CI: 95% confidence interval.Source: Elaborated by the authors.

To further elucidate the relationship between the NLR and PLR with CA, we conducted both univariate and multivariate logistic regression analyses. In the univariate analysis, factors such as patient age, onset time, peritonitis, neutrophil count, lymphocyte count, NLR, and PLR showed associations with CA. However, after adjusting for confounders in the multivariate analysis, only the onset time (*odds ratio* [OR] = 1.04, 95%CI 1.03–1.06, *p* < 0.001) and NLR (OR = 0.31, 95%CI 0.11–0.84, *p* = 0.021) remained as independent risk factors for CA, whereas PLR did not (OR = 0.50, 95%CI 0.24–1.06, *p* = 0.068) (Table 4). Figure 3 illustrates the relationship between NLR, PLR, and the incidence of CA.

**Table 4:** Univariate and multivariate analysis (excluding combined neutrophil-platelet score).

Characteristics	Univariate		*p* -value	Multivariate		*p* -value
	OR	95%CI		OR	95%CI	
Age	1.03	1.01–1.05	0.003	1.02	0.99–1.04	0.13
Onset time	1.03	1.02–1.05	< 0.001	1.04	1.03–1.06	< 0.001
Peritonitis						
No	Ref.	Ref.	Ref.	Ref.	Ref.	Ref.
Right lower abdomen	1.16	0.6–2.38	0.672	1.42	0.67–3.16	0.37
Lower abdomen	3.78	1.15–12.82	0.028	2.42	0.56–10.45	0.23
Whole abdomen	1.5	0.41–5	0.514	0.97	0.23–3.8	0.97
NEU	1.14	1.07–1.23	< 0.001	1.09	0.99–1.21	0.07
LYM	0.5	0.34–0.72	< 0.001	1.27	0.69–2.11	0.39
NLR	0.23	0.13–0.4	< 0.001	0.31	0.11–0.84	0.02
PLR	0.31	0.18–0.51	< 0.001	0.5	0.24–1.06	0.07
PLA	1	1–1.01	0.33			
Gender						
female	Ref.	Ref.	Ref.			
male	1.11	0.68–1.82	0.68			
Smoking status						
Nonsmoker	Ref.	Ref.	Ref.			
Previous smokers	0.5	0.22–1.03	0.08			
Occasional smokers	0.68	0.1–2.89	0.64			
Regular smokers	0.53	0.08–2.11	0.42			
Alcohol intake						
Nondrinker	Ref.	Ref.	Ref.			
Previous drinkers	0.19	0.01–0.96	0.11			
Occasional drinkers	0.68	0.33–1.32	0.27			
Regular drinkers	1.21	0.06–12.84	0.88			

NEU: neutrophils; LYM: lymphocyte; NLR: neutrophil-to-lymphocyte ratio; PLA: platelet; PLR: platelet-lymphocyte ratio; OR: *odds ratio*; 95%CI: confidence interval. Source: Elaborated by the authors.

To enhance the predictive accuracy of inflammatory markers for CA, we developed a parameter called the combined neutrophil-platelet score (CNPS), based on methods suggested by Miao et al.^16^ and Huang et al.^17^. CNPS combines both NLR and PLR, and is calculated as Eq. 3:


(3) CNPS=NLR+PLR

If NLR ≥ 6.45, then it scores 1, otherwise 0; similarly, if PLR ≥ 177.365, then it scores 1, otherwise 0. We evaluated the diagnostic performance of CNPS by plotting the ROC curve and calculating the AUC, which was found to be 0.688 (95%CI 0.63–0.75) (Fig. 2). This performance was superior to both individual NLR and PLR scores. Details regarding the sensitivity, specificity, and other parameters of CNPS can be found in Table 2. Furthermore, we analyzed patient data, categorizing it into high and low CNPS groups based on a cutoff value of 0.5. The high CNPS group displayed a significantly higher percentage of patients with CA, older age, and more severe cases featuring both lower abdominal and total peritonitis (Table 3). Multivariate analysis, controlling for confounders, confirmed that CNPS is an independent risk factor for CA, with an OR of 2.52 (95%CI 1.81–3.58, *p* < 0.001) (Table 5).

**Table 5:** Univariate and multivariate analysis (including combined neutrophil-platelet score).

Characteristics	Univariate		*p* -value	Multivariate		*p* -value
	OR	95%CI		OR	95%CI	
Age	1.03	1.01–1.05	0.003	1.02	0.99–1.04	0.15
Onset time	1.03	1.02–1.05	p < 0.001	1.04	1.02–1.05	p < 0.001
Peritonitis						
No	Ref.	Ref.	Ref.	Ref.	Ref.	Ref.
Right lower abdomen	1.16	0.6–2.38	0.67	1.58	0.76–3.47	0.24
Lower abdomen	3.78	1.15–12.82	0.03	2.69	0.64–11.25	0.17
Whole abdomen	1.5	0.41–5	0.51	0.97	0.23–3.64	0.96
CNPS	2.31	1.71–3.17	p < 0.001	2.52	1.81–3.58	p < 0.001
Gender						
female	Ref.	Ref.	Ref.			
male	1.11	0.68–1.82	0.68			
Smoking status						
Nonsmoker	Ref.	Ref.	Ref.			
Previous smokers	0.5	0.22–1.03	0.08			
Occasional smokers	0.68	0.1–2.89	0.64			
Regular smokers	0.53	0.08–2.11	0.42			
Alcohol intake						
Nondrinker	Ref.	Ref.	Ref.			
Previous drinkers	0.19	0.01–0.96	0.11			
Occasional drinkers	0.68	0.33–1.32	0.27			
Regular drinkers	1.21	0.06–12.84	0.88			

CNPS: combined neutrophil-platelet score; OR: *odds ratio*; 95%CI: 95% confidence interval. Source: Elaborated by the authors.

In the prospective study, a total of 292 patients clinically diagnosed with acute appendicitis were enrolled. However, one patient was intraoperatively diagnosed with gastric perforation, another with intestinal obstruction, and one was postoperatively diagnosed with a low-grade appendiceal mucinous neoplasm. Sixteen patients declined surgery after enrollment and were discharged following successful non-operative management. An additional nine patients voluntarily withdrew from the study for personal reasons. After excluding these 28 cases, 264 patients were included in the final analysis, comprising 114 males and 150 females, with a median age of 44 years old (32–53). The diagnostic performance of the novel inflammatory marker, CNPS, was evaluated using ROC curve analysis. The AUC was 0.707 (95%CI 0.645–0.767), with a sensitivity of 0.809 (95%CI 0.727–0.891), specificity of 0.606 (95%CI 0.533–0.678), positive predictive value of 0.511 (95%CI 0.428–0.593), and negative predictive value of 0.862 (95%CI 0.801–0.923).

## Discussion

The annual incidence of acute appendicitis is estimated to range from 96.5 to 100 cases per 100,000 adults^18^, with the highest occurrence in individuals aged 10 to 30 years old^19^. Rajalingam et al.^20^ found that individuals have a 7% lifetime risk of developing appendicitis.

Livingston et al.^21^ reported that appendiceal perforation occurs in approximately 16–40% of acute appendicitis cases. This complication is more common among young adults and those over 50 years old. Cruz-Vallejo et al.^22^ noted a higher incidence of appendiceal perforation in male patients compared to females. In our study, the mean age of patients with CA was 45 years old, whereas the mean age for those with UA was 40, a difference that was observed between the two groups. We did not find any significant difference between male and female patients in the CA group.

Various laboratory markers have been utilized to diagnose appendicitis and evaluate its severity. Leukocyte elevation, for instance, is often observed in the early stages of acute appendicitis^23^. Yang et al.^24^ reported that leukocyte elevation has a sensitivity of 87.2% and specificity of 33.1% for diagnosing acute appendicitis. However, it does not effectively predict the severity of the condition^25^. C-reactive protein (CRP) also increases during inflammation. One study^26^ indicated that CRP has a positive predictive value of 94.7%, with a sensitivity of 85.1% and specificity of 72% for diagnosing acute appendicitis. Nonetheless, CRP is not a reliable marker for assessing inflammation severity. Rajalingam et al.^20^ showed CRP with a sensitivity of only 74.7% and specificity of 40.7%. Although several studies^27^ have associated elevated serum bilirubin with appendiceal perforation, its sensitivity and specificity are insufficient. Consequently, surgeons continually seek a non-invasive, reliable tool or marker with high sensitivity and specificity to predict the severity of appendicitis.

NLR provides insights into two distinct inflammatory pathways, making it a potential predictor of acute appendicitis and its severity. The neutrophil count reflects the active and persistent inflammatory state, while the lymphocyte count highlights regulatory pathways^28^. A study by Jung et al.^29^ demonstrated that NLR predicted appendiceal perforation with a sensitivity of 78% and specificity of 66%. Similarly, Pehlivanlı et al.^30^ reported a sensitivity of 64% and specificity of 64%. In our study, NLR was higher in the CA group compared to the UA group. Moreover, using NLR ≥ 6.45 to predict CA yielded an AUC of 0.675 (95%CI 0.61–0.74), with a sensitivity of 78.2% (95%CI 0.69–0.87) and specificity of 54.9% (95%CI 0.49–0.61). After controlling for confounders, NLR remained an independent risk factor for CA.

The study by Pehlivanlı et al.^30^ determined that acute appendicitis could be diagnosed with a PLR cutoff value of 140.45, achieving a sensitivity of 71.4% and specificity of 88.9%. However, distinguishing between UA and CA required a PLR cutoff of 163.27, with a sensitivity of 64.3% and specificity of 67.5%. Rajalingam et al.^20^ found that a PLR > 154.98 indicated UA (sensitivity = 75.9%, specificity = 40.8%), whereas a PLR > 180.5 suggested CA (sensitivity = 22.4%, specificity = 89%). In our study, PLR values were higher in the CA group compared to the UA group (*p* < 0.05). With a PLR ≥ 177.37, the AUC was 0.652 (95%CI 0.59–0.72), sensitivity was 59.8% (95%CI 0.49–0.7), and specificity was 68.5% (95%CI 0.63–0.74). Univariate analysis indicated an association between PLR and CA, but multivariate analysis did not confirm PLR as an independent risk factor for CA.

After our analysis, we found that both NLR and PLR exhibited good predictive abilities, with AUCs of 0.675 and 0.652, respectively. However, NLR demonstrated high sensitivity (0.782) but poor specificity (0.549), whereas PLR had higher specificity (0.685) but slightly lower sensitivity (0.598). Furthermore, after controlling for confounders, we determined that only NLR was an independent risk factor for CA, while PLR was not. Consequently, we integrated NLR and PLR into a new inflammatory parameter: CNPS. The diagnostic and predictive abilities of CNPS surpassed those of NLR and PLR, with an AUC of 0.688 (95%CI 0.63–0.75) and a higher sensitivity (0.816). Additionally, univariate and multivariate analyses confirmed that CNPS was an independent risk factor for CA.

In the prospective study, we further validated the clinical utility of the CNPS. Although 292 patients with a clinical diagnosis of acute appendicitis were initially enrolled, only 264 cases were ultimately included in the final analysis.

ROC curve analysis demonstrated that CNPS had good diagnostic performance for complicated appendicitis, with AUC of 0.707 (95%CI 0.645–0.767) and sensitivity of 80.9%. These findings support the practical value of CNPS in clinical settings. One possible explanation is that CNPS integrates both the NLR and the PLR, thereby offering superior diagnostic performance compared to individual markers. This combined index may help clinicians more effectively identify high-risk patients with CA at an early stage and facilitate timely intervention.

There are noteworthy limitations to this study, and caution remains necessary in interpreting this new marker. First, we only explored the value of this marker in adult patients with appendicitis; further studies are needed in pediatric and elderly patients. Second, the sample size in both the retrospective and prospective parts of our study was relatively limited. Third, inflammatory markers may fluctuate during hospitalization. Although we mitigated confounding effects by using baseline levels at admission, we did not analyze changes throughout hospitalization. Fourth, the specificity of the new inflammatory parameters could be improved. Moreover, the construction of CNPS was a posteriori, *i.e.*, it was developed after finding that neither NLR nor PLR alone had satisfactory diagnostic performance for CA. The cut-off values were derived from the retrospective cohort in an exploratory manner, which may carry a risk of overfitting. The prospective validation partly mitigates this concern, but further external validation is needed.

## Conclusion

In this study, we found that NLR was an independent risk factor for CA, whereas PLR was not. Consequently, we combined NLR and PLR into a new inflammatory index: CNPS. We systematically evaluated the practical value of CNPS in diagnosing and predicting CA. A multicenter prospective study with a sufficiently large and diverse sample size is necessary to further validate these findings in the future.

## Data Availability

The data that support the findings of this study are available from the corresponding author upon reasonable request.
